# Clinicopathologic Features and Postoperative Outcomes of Parotidectomy: A 16-Year Retrospective Cohort Study from a Tertiary Referral Center

**DOI:** 10.3390/diagnostics16020216

**Published:** 2026-01-09

**Authors:** Seval Akay, Ozlem Yagiz Agayarov, Volkan Semiz, Ulku Kucuk, Ilker Burak Arslan, Olcun Umit Unal, Ibrahim Cukurova

**Affiliations:** 1 Medical Oncology Department, Izmir City Hospital, Izmir 35400, Türkiye; 2 Head and Neck Department, Izmir Tepecik and Education Hospital, Izmir 35020, Türkiye; drozlemyagizagayarov@gmail.com (O.Y.A.);; 3 Radiation Oncology Department, Izmir City Hospital, Izmir 35400, Türkiye; 4 Pathology Department, Izmir City Hospital, Izmir 35400, Türkiye; 5 Head and Neck Department, Izmir City Hospital, Izmir 35400, Türkiye

**Keywords:** parotid neoplasms, parotidectomy, facial paralysis, neoplasm invasiveness, histology

## Abstract

**Background**: Parotid gland tumors pose diagnostic and surgical challenges due to their histological heterogeneity and proximity to the facial nerve. This study aimed to evaluate clinicopathological features and postoperative outcomes with a specific focus on facial nerve function in patients undergoing parotidectomy. **Methods**: This retrospective study included 314 patients who underwent parotidectomy between 2008 and 2024 at a tertiary center. Demographic data, tumor histology, and postoperative complications—particularly facial nerve paralysis within the first three months—were analyzed. Histopathological features including capsular, perineural, and lymphovascular invasion were also assessed. **Results**: Of all cases, 79% were benign, 14.6% malignant, and 6.4% non-neoplastic. Pleomorphic adenoma and Warthin tumor were the most common benign entities, while mucoepidermoid carcinoma was the most frequent malignancy. Malignant tumors were associated with higher rates of positive surgical margins (44.2% vs. 12.5%, *p* < 0.001), capsular invasion (25% vs. 7%, *p* < 0.001), and tumor necrosis (22% vs. <1%, *p* < 0.001). Facial paralysis occurred in 4.4% of patients, largely transient and significantly associated with malignant tumors (*p* < 0.001) and extensive lymph node dissection (*p* < 0.001). Capsular invasion and necrosis were rare in benign lesions but still observed, especially in pleomorphic adenoma. **Conclusions**: Histopathologic aggressiveness markers were associated with malignant disease and postoperative facial nerve dysfunction. These findings support a risk-stratified approach to follow-up: all patients undergo universal early assessment at two weeks and three months, after which surveillance intensity may be individualized according to histopathologic features—such as necrosis, perineural invasion, capsular invasion, or positive margins.

## 1. Introduction

Parotid gland lesions have long posed diagnostic and therapeutic challenges due to their histological diversity and close anatomical proximity to the facial nerve. They encompass a broad etiological spectrum, including benign and malignant neoplasms, inflammatory conditions, and obstructive pathologies such as sialolithiasis [1]. Parotid tumors are rare entities, accounting for approximately 3% of all head and neck tumors [2]. Clinically, they often present as preauricular masses, with approximately three-quarters being benign and less than one-quarter malignant [3,4].

The most common benign tumor of the parotid gland is pleomorphic adenoma, followed by Warthin tumor, while mucoepidermoid carcinoma is the most frequent malignant entity. Among non-neoplastic lesions, inflammatory conditions are predominant [5,6].

Diagnostic evaluation typically utilizes ultrasound (US), magnetic resonance imaging (MRI), and computed tomography (CT), complemented by fine-needle aspiration biopsy (FNAB). However, given the limited sensitivity and specificity of these modalities, histopathological examination remains the gold standard for definitive diagnosis [7].

Surgical excision is the cornerstone of treatment for parotid tumors. Nevertheless, parotidectomy poses considerable technical challenges due to the intricate anatomical relationship between the gland and the facial nerve. The branching of the nerve within the gland increases the risk of iatrogenic injury, potentially resulting in functional and aesthetic deficits such as facial paralysis.

This retrospective study seeks to provide insight into clinicopathological patterns and surgical outcomes following parotidectomy, emphasizing facial nerve complications and histopathological diversity in relation to histopathologic aggressiveness markers (capsular invasion, necrosis, perineural and lymphovascular invasion). It also describes a subgroup of patients without neoplastic lesions on final histopathology, highlighting limitations of preoperative diagnostics and the risk of overtreatment.

## 2. Materials and Methods

This retrospective observational study included 314 patients who underwent parotidectomy for a parotid mass between 2008 and 2024 at Izmir Tepecik Training and Research Hospital, a tertiary care center. All procedures were performed by a dedicated head and neck surgical team with substantial experience in parotid gland surgery.

Pediatric patients (<18 years) were included only if a parotidectomy was performed and histopathology was available. Recurrent tumors and parotid metastases from cutaneous malignancies (e.g., SCC, melanoma) were included, as they represent clinically relevant parotid disease. Exclusion criteria comprised incomplete medical or pathological records and revision surgeries for recurrences of procedures originally performed at external institutions.

Superficial parotidectomy was performed for lesions confined to the superficial lobe, while total parotidectomy was undertaken when tumors involved or were suspected to involve the deep lobe. Deep lobe involvement on imaging was defined as extension medial to the plane of the facial nerve and/or radiologic contact with the parapharyngeal space. Facial nerve preservation was considered in all cases unless oncologic clearance required the sacrifice of a branch. Intraoperative identification of the facial nerve trunk was routinely performed using anatomical landmarks, including the tragal pointer and posterior belly of the digastric muscle. Elective neck dissection was performed according to predefined clinical criteria, including preoperative suspicion of malignancy, high-grade FNAB findings, advanced tumor size, radiologic suspicion of nodal involvement, and histologic subtypes associated with high risk of occult metastasis.

Histopathologic diagnoses were performed according to routine diagnostic practice consistent with the WHO Classification of Head and Neck Tumours (4th edition, 2017) [8], which served as the standard reference during the majority of the study period. Since data collection began in 2008, earlier cases were diagnosed according to the prevailing diagnostic conventions of that time, with terminology and criteria later aligned to WHO-recognized entities where feasible. Representative recent cases were reviewed, considering the 2022 WHO updates to ensure consistency with contemporary classification. Immunohistochemical and molecular studies were not uniformly available across the cohort, preventing consistent molecular confirmation of specific tumor subtypes (such as CRTC1-MAML2, MYB–NFIB, or PLAG1/HMGA2 rearrangements) and therefore precluding genotype–phenotype correlation analysis.

Data were retrieved from electronic medical records and included demographic details (e.g., age, sex), tumor characteristics (laterality and histopathological classification as non-neoplastic, benign, or malignant), and histological features such as necrosis, capsular invasion, lymphovascular invasion, and perineural invasion. Tumor terminology and variant classification were retained as originally reported in the pathology records, as variant-level subclassification was not consistently available throughout the retrospective period. In keeping with the retrospective nature of the study, all tumor diagnoses were taken directly from the original pathology reports of their respective dates, reflecting the diagnostic standards, terminology, and ancillary tools available at the time of interpretation.

Postoperative complications, specifically facial nerve paralysis, were documented based on facial nerve motor function. All patients were clinically evaluated at 2 weeks postoperatively and again at 3 months following surgery to assess wound healing, facial nerve function, and early complications. The primary postoperative outcome was early surgical morbidity, defined as the presence of new-onset facial nerve paralysis documented during the immediate postoperative period and follow-up visits at our center. Long-term oncologic outcomes (recurrence, residual disease, and reoperation for tumor) were not systematically available for all patients because many were referred back to peripheral institutions after surgery.

Histopathological diagnoses were based on postoperative tissue analyses conducted by experienced pathologists. In cases of diagnostic ambiguity, specimens were re-evaluated by a senior pathologist to ensure consistency and diagnostic accuracy.

All statistical analyses were performed using IBM SPSS Statistics, Version 20.0 (IBM Corp., Armonk, NY, USA). Descriptive statistics are reported as mean ± standard deviation (SD), as well as minimum and maximum values. The Kolmogorov–Smirnov test was used to assess the normality of data distribution. For normally distributed continuous variables, comparisons between two groups were performed using the independent samples *t*-test, and comparisons among more than two groups were analyzed using one-way ANOVA. When the assumption of normality was violated, the Kruskal–Wallis test was used for comparisons among three or more groups, and the Mann–Whitney U test for two-group comparisons. For categorical variables, the Chi-square test was applied. A *p*-value < 0.05 was considered statistically significant.

Ethical approval was obtained from the local institutional review board of Tepecik Education and Research (protocol code: 2024/06-02, approval date: 2 July 2024). Data collection and retrospective evaluation were limited to 2024, as members of the research team subsequently relocated to other institutions, preventing extended data capture beyond that date. This study was conducted in accordance with the principles of the Declaration of Helsinki.

## 3. Results

### 3.1. Patients’ Demographics

**Figure 1:**
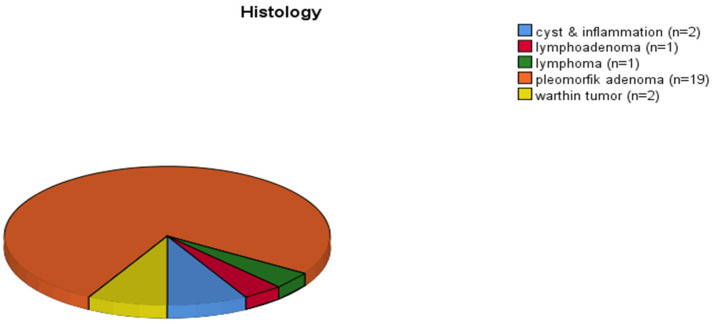
Distribution of histologic subtypes in patients under 35 years.

Of the 314 patients included in the study, 53% were male and 47% female, with a mean age of 55 ± 13.45 years (range: 10–88 years. Pediatric patients (*n* = 5) uniformly presented with pleomorphic adenoma. Among patients under 35 years old (*n* = 25), pleomorphic adenoma was the most frequently observed histological type (Figure 1).

### 3.2. Histological Classification

Histopathological classification revealed that 79% of the parotid lesions were benign, 14.6% were malignant, and 6.4% were non-neoplastic. Among benign tumors, pleomorphic adenoma was the most common (*n* = 92), while mucoepidermoid carcinoma (*n* = 17; 11 low-grade, 6 high-grade) was the predominant malignant tumor. Pleomorphic adenoma, basal cell monomorphic adenoma, and low-grade mucoepidermoid carcinoma were more frequently observed in female patients, with female-to-male ratios of 3:2, 3:1, and 3:1, respectively. In contrast, Warthin tumor was more prevalent among males, with a male-to-female ratio of 2:1. The detailed distribution of histopathological subtypes is presented in Table 1.

### 3.3. Tumor Biology and Histopathologic Features

The pathological diagnoses were classified into three groups: malignant, benign, and non-neoplastic, as summarized in Table 2. While mean age did not differ significantly between the malignant group and the benign (*p* = 0.561) or non-neoplastic (*p* = 0.193) groups, patients with non-neoplastic lesions were significantly older than those with benign tumors (*p* = 0.047). No significant differences were observed in terms of lesion laterality or sex distribution among the three groups (*p* = 0.317 and *p* = 0.871, respectively).

Among the sixteen benign cases showing capsular invasion, thirteen were diagnosed as pleomorphic adenoma, three as Warthin tumor, and one as oncocytoma. None of these patients required additional treatment, and close clinical follow-up was scheduled. Conversely, adjuvant radiotherapy was administered to eleven patients with malignant tumors that demonstrated capsular invasion. The incidence of capsular invasion was significantly higher in malignant tumors compared to benign ones (25% vs. 7%, *p* < 0.001).

Lymphovascular invasion was noted in a single benign tumor diagnosed as a basal cell monomorphic adenoma, while perineural invasion was observed in one pleomorphic adenoma case.

Tumor necrosis was identified in twenty cases. Among these, one was observed in granulomatous inflammation of non-neoplastic parotid tissue, and another in a benign pleomorphic adenoma. Necrosis was reported in ten malignant tumors, five of which were high-grade mucoepidermoid carcinomas. The remaining five consisted of adenoid cystic carcinoma, alveolar soft part sarcoma, basal cell carcinoma, ductal-type carcinoma, and carcinoma ex pleomorphic adenoma—subtypes known for their aggressive clinical behavior. The presence of tumor necrosis was significantly more common in malignant tumors (*p* < 0.001) and was interpreted as an indicator of their aggressive biological nature.

### 3.4. Surgical Outcomes

All non-neoplastic lesions were resected with clear (R0) surgical margins. In contrast, positive surgical margins (R1) were significantly more frequent in malignant tumors (44.2%) compared to benign lesions (12.8%) (*p* < 0.001). Among the patients with R1 resection, nine were diagnosed with mucoepidermoid carcinoma, two with adenoid cystic carcinoma, two with melanoma, two with carcinoma ex pleomorphic adenoma, and the remaining with basal cell carcinoma, ductal-type carcinoma, epithelial carcinoma, and lymphoepithelial carcinoma (one case each). Among benign tumors with R1 resection, twenty were pleomorphic adenomas, ten were Warthin tumors, and one was diagnosed as myoepithelioma. No R1 resections were identified in non-neoplastic lesions. Overall, the R1 resection rate in the study cohort was 16.3%.

### 3.5. Postoperative Complications-Facial Nerve Dysfunction

Facial paralysis within the first three months postoperatively was observed in fourteen patients (4.4%), equally distributed by sex (7 males, 7 females). Of these, ten had malignant tumors, and three had benign lesions. One notable case involved a 56-year-old woman with a prior history of breast cancer who underwent right parotidectomy following a fine needle aspiration biopsy suggestive of Warthin tumor. Postoperatively, she developed stage 4 facial paralysis. During lymph node dissection, 25 lymph nodes were removed, all of which were histopathologically reactive. Among patients without facial paralysis, the median number of lymph nodes removed was 0 (mean: 2.5), whereas in those with facial paralysis, the median was 17 (mean: 18). This difference was statistically significant (*p* < 0.001).

A total of six patients underwent parotidectomy, and the pathological examination revealed only normal parotid tissue. Clinical details of these patients are summarized below:

• Patient 1: A 68-year-old male who underwent parotidectomy during evaluation of a cervical lymph node recurrence 19 months after excision of a squamous cell carcinoma (SCC) from the left temporal region.
• Patient 2: A 71-year-old male who underwent parotidectomy during treatment for SCC of the right auricle.
• Patient 3: A 70-year-old female who had a parotidectomy simultaneously with excision of SCC of the right external auditory canal.
• Patient 4: A 60-year-old male with two consecutive nondiagnostic FNAB of the parotid.
• Patient 5: A 45-year-old male whose FNAB was interpreted as favoring a low-grade salivary gland neoplasm.
• Patient 6: A 38-year-old female with an FNAB suggestive of pleomorphic adenoma.

## 4. Discussion

The clinical evaluation of parotid lesions remains challenging due to their heterogeneous histopathology and overlapping presentations. FNAC serves as a widely used initial diagnostic tool, but its sensitivity—particularly in differentiating benign from low-grade malignant tumors—is limited, as demonstrated in multiple cohort analyses. Therefore, histopathological examination remains the definitive diagnostic standard [9].

Pleomorphic adenoma, followed by Warthin tumor, were the most common benign tumors in our cohort, consistent with global distributions [10,11,12]. Although benign, these lesions warrant surgical excision due to their potential for recurrence or malignant transformation [13]. Adjuvant treatment is generally reserved for cases demonstrating adverse pathological features such as capsular invasion, perineural invasion (PNI), nodal metastasis, or positive margins [14,15], while chemotherapy plays a minor role and is typically limited to advanced disease [16].

Among histopathologic markers, necrosis, PNI, and capsular invasion emerged as indicators of more aggressive behavior, in agreement with previous observations [17,18,19,20,21,22]. Importantly, a subset of benign lesions (e.g., pleomorphic adenoma) demonstrated aggressive markers such as capsular invasion, necrosis, or PNI, suggesting that histologic risk features may supersede conventional benign–malignant distinctions in shaping follow-up strategies. The presence of these features should therefore be incorporated into postoperative risk stratification. Additionally, nodal metastasis is strongly associated with poorer survival and appears more common in high-grade subtypes such as salivary duct carcinoma and high-grade mucoepidermoid carcinoma [23,24]. While we observed an association between extensive nodal dissection and transient postoperative facial nerve dysfunction, this likely reflects underlying tumor aggressiveness and anatomical complexity rather than a direct causal effect of lymphadenectomy [25].

Positive surgical margins (R1 resection) are well-recognized as an adverse prognostic factor associated with poorer survival outcomes in malignant cases; however, these patients demonstrate a favorable response to adjuvant radiotherapy [20,21,26]. Our data support the view that benign tumors with positive margins—in the absence of necrosis, PNI, or capsular invasion—may be safely managed with observation. However, the presence of these aggressive features should prompt consideration for re-excision or closer imaging-based follow-up.

Collectively, these findings advocate for a risk-stratified postoperative surveillance strategy. We recommend clinical follow-up every 6–12 months for low-risk benign tumors lacking adverse histopathologic features. Conversely, high-risk benign tumors—defined by the presence of necrosis, capsular, or perineural invasion (PNI)—and all malignant lesions warrant intensified monitoring every 3–6 months, with adjunctive imaging as indicated.

Regarding benign tumors with R1 margins, those without aggressive markers may be safely managed with observation; however, the presence of such markers should prompt consideration for re-excision or stricter surveillance due to elevated recurrence risk. For malignant tumors, adjuvant radiotherapy remains indicated in the presence of adverse features—such as positive margins, lymphovascular invasion, and nodal involvement—where it has been shown to improve survival outcomes and locoregional control.

This study has several limitations. First, its retrospective nature may have introduced selection bias, particularly regarding the indication for surgery and the completeness of histopathological records. Second, the single-center design limits the generalizability of our findings to broader populations with different demographic or healthcare characteristics. Third, systematic long-term follow-up data on recurrence, reoperation, and survival were not available for all patients, which precludes a robust analysis of oncologic outcomes and limits our ability to correlate histopathologic risk factors with definitive endpoints. As a result, our conclusions primarily relate to early postoperative surgical outcomes and do not allow the validation of specific risk-adapted surveillance schedules. Future prospective multicenter studies with standardized follow-up protocols are necessary to validate these observations and further refine treatment and follow-up strategies.

## 5. Conclusions

This 16-year retrospective study provides a comprehensive overview of the demographic and clinicopathological characteristics of patients undergoing parotidectomy at a tertiary center. The majority of cases were benign, with pleomorphic adenoma and Warthin tumor being the most common. Malignant tumors made up 14.6% of cases and showed more aggressive features such as higher rates of positive surgical margins, capsular invasion, and tumor necrosis.

Our results suggest that histopathologic aggressiveness markers, even in benign tumors, may warrant more extensive surgery and closer postoperative follow-up. These markers were also associated with malignant disease. The observed association between nodal dissection and transient facial nerve dysfunction in this cohort should be considered correlational rather than causative, given the influence of tumor aggressiveness and the limitations of retrospective data. In our institution, all patients undergo routine follow-up at two weeks and three months, after which surveillance intensity is individualized according to risk factors—such as tumor necrosis, perineural invasion, capsular invasion, lymphovascular invasion, and positive surgical margins. Positive margins were most frequent in mucoepidermoid carcinoma and carcinoma ex pleomorphic adenoma, supporting consideration of postoperative radiotherapy in these histologic subtypes. Taken together, these pathological features should guide the extent of surgery, the need for adjuvant treatment, and the frequency of clinical and imaging surveillance, while recognizing that our retrospective dataset does not allow the definition of rigid or universally applicable follow-up protocols.

## Figures and Tables

**Table 1:** Distribution of histopathological diagnoses by benign, malignant, and non-neoplastic characteristics (Percentages in parentheses belong to columns). SCC: squamous cell carcinoma.

Malignant (*n* = 46)	Benign (*n* = 248)	Non-Neoplastic (*n* = 20)
Adenoid Cystic Carcinoma (6.5%)	Basal Cell Monomorphic Adenoma (4.8%)	Acute Suppurative Sialadenitis (5%)
Alveolar Soft Part Sarcoma (2.2%)	Lymphadenoma (<1%)	Granulomatous Tissue (5%)
Acinic Cell Carcinoma (6.5%)	Lymphoepithelioma (<1%)	Cyst and Inflammation (25%)
Basal Cell Carcinoma (4.3%)	Monomorphic Adenoma (<1%)	Chronic Sialadenitis (20%)
Ductal Type Carcinoma (4.3%)	Myoepithelioma (1.6%)	Lymphoepithelial Cyst (10%)
Epithelial Carcinoma (2.2%)	Oncocytoma (<1%)	Lipoma (5%)
Carcinoma ex Pleomorphic Adenoma (10.9%)	Pleomorphic Adenoma (37.1%)	Normal Parotid Tissue (30%)
Lymphoepithelial Carcinoma (4.3%)	Warthin Tumor (54.4%)	
Lymphoma (10.9%)		
Malignant Oncocytoma (2.2%)		
Melanoma (6.5%)		
Mucoepidermoid Carcinoma-High Grade (13.1%)		
Mucoepidermoid Carcinoma-Low Grade (23.9%)		
SCC—Fistulized to Skin (2.2%)		

**Table 2:** Comparison of clinicopathological features among malignant, benign, and non-neoplastic parotid gland lesions.

Parameter	Malignant (*n* = 46)	Benign (*n* = 248)	Non-Neoplastic (*n* = 20)
Median Age (±SD)	56.74 ± 12.88	53.02 ± 13.21	60.40 ± 15.66
Laterality *			
Right	63%	52.0%	55%
Left	34.7%	47.6%	45%
Sex			
Female	50%	46.4%	50%
Male	50%	53.6%	50%
Surgical Margins *			
R0 (clear)	52.2%	85.5%	100%
R1 (positive)	41.3%	12.5%	0%
Capsular Invasion *			
Present	23.9%	6.4%	0%
Absent	71.7%	92.3%	100%
Lymphovascular Invasion *			
Present	39.1%	<1%	0%
Absent	58.7%	98.8%	100%
Perineural Invasion *			
Present	36.9%	<1%	0%
Absent	60.9%	99.2%	100%
Tumor Necrosis *			
Present	21.7%	<1%	5%
Absent	70.1%	99.2%	95%
Facial Paralysis *			
Present	21.7%	1.2%	5%
Absent	78.3%	98.8%	95%

* If there is missing data, the total might not be 100% in the columns.

## Data Availability

The data presented in this study are available on reasonable request from the corresponding author. The data are not publicly available due to institutional privacy regulations.
